# Supplementary material for: Comparative multiomics analysis of cell physiological state after culture in a basket bioreactor
Source: Sci Rep. 2022 Nov 23;12:20161. doi: 10.1038/s41598-022-24687-4 (PMC9686226; doi:10.1038/s41598-022-24687-4)
Supplement: Supplementary file 1 — Supplementary Information 1. [file 41598_2022_24687_MOESM1_ESM.zip › raw data/Transcriptomic raw data/6.Enrichment/1.GO/TE1vsTC1/GSEA/heat_map_corr_plot.html]

Heat map and correlation plot for readcount  

Fig 1: heat\_map      
 Heat Map of the top 50 features for each phenotype in readcount

  
  

Fig 2: Ranked Gene List Correlation Profile      
 Ranked list correlations for readcount

  
  
    
